# Computer-Aided Diagnosis and Localization of Lateralized Temporal Lobe Epilepsy Using Interictal FDG-PET

**DOI:** 10.3389/fneur.2013.00031

**Published:** 2013-04-03

**Authors:** Wesley T. Kerr, Stefan T. Nguyen, Andrew Y. Cho, Edward P. Lau, Daniel H. Silverman, Pamela K. Douglas, Navya M. Reddy, Ariana Anderson, Jennifer Bramen, Noriko Salamon, John M. Stern, Mark S. Cohen

**Affiliations:** ^1^Department of Biomathematics, David Geffen School of Medicine, University of California Los AngelesLos Angeles, CA, USA; ^2^Laboratory of Integrative Neuroimaging Technology, Department of Psychiatry, Neuropsychiatric Institute, University of California Los AngelesLos Angeles, CA, USA; ^3^Ahmanson Translational Imaging Division, Department of Molecular and Medical Pharmacology, David Geffen School of Medicine, University of California Los AngelesLos Angeles, CA, USA; ^4^Department of Neurology, Seizure Disorder Center, University of California Los AngelesLos Angeles, CA, USA; ^5^Laboratory of Integrative Neuroimaging Technology, Departments of Psychiatry, Neurology, Radiology, Biomedical Physics, Psychology and Bioengineering, University of California Los AngelesLos Angeles, CA, USA

**Keywords:** epilepsy, computer-aided diagnosis, mutual information, temporal lobe epilepsy, PET, fluoro-deoxyglucose positron emission tomography, machine learning

## Abstract

Interictal FDG-PET (iPET) is a core tool for localizing the epileptogenic focus, potentially before structural MRI, that does not require rare and transient epileptiform discharges or seizures on EEG. The visual interpretation of iPET is challenging and requires years of epilepsy-specific expertise. We have developed an automated computer-aided diagnostic (CAD) tool that has the potential to work both independent of and synergistically with expert analysis. Our tool operates on distributed metabolic changes across the whole brain measured by iPET to both diagnose and lateralize temporal lobe epilepsy (TLE). When diagnosing left TLE (LTLE) or right TLE (RTLE) vs. non-epileptic seizures (NES), our accuracy in reproducing the results of the gold standard long term video-EEG monitoring was 82% [95% confidence interval (CI) 69–90%] or 88% (95% CI 76–94%), respectively. The classifier that both diagnosed and lateralized the disease had overall accuracy of 76% (95% CI 66–84%), where 89% (95% CI 77–96%) of patients correctly identified with epilepsy were correctly lateralized. When identifying LTLE, our CAD tool utilized metabolic changes across the entire brain. By contrast, only temporal regions and the right frontal lobe cortex, were needed to identify RTLE accurately, a finding consistent with clinical observations and indicative of a potential pathophysiological difference between RTLE and LTLE. The goal of CADs is to *complement* – not replace – expert analysis. In our dataset, the accuracy of manual analysis (MA) of iPET (∼80%) was similar to CAD. The square correlation between our CAD tool and MA, however, was only 30%, indicating that our CAD tool does not recreate MA. The addition of clinical information to our CAD, however, did not substantively change performance. These results suggest that automated analysis might provide clinically valuable information to focus treatment more effectively.

## Introduction

It is difficult to differentiate between patients with epilepsy (PWE), and those with non-epileptic seizures (NES). The clinical assessment relies on the report of untrained witnesses or the patients themselves. A non-epileptic seizure is defined as the presence of external seizure symptoms and/or signs with no electrographic features characteristic of epilepsy. Long term video-EEG monitoring has shown consistently that roughly one third of patients diagnosed with “medication refractory epilepsy” in fact suffer from NES (Kerr et al., 2012a). Because they don’t suffer from epilepsy, these patients with NES (PWN) are not treated effectively with anti-epileptic drugs (AEDs). For the majority of PWN, the NES are a manifestation of dissociative or conversion disorder in which their psychological challenges manifest themselves physically (Marchetti et al., 2008, 2009). A minority of PWN suffers from organic, non-epileptic maladies that can be confused with seizure disorder including, but not limited to, dementia and cardiovascular disease (Sahaya et al., 2011). The gold standard for the differential diagnosis and pre-surgical assessment of epilepsy includes 72 or more hours of video-EEG monitoring (Cragar et al., 2002; LaFrance and Devinsky, 2004). However, 10% of patients admitted for this extensive assessment leave with inconclusive results (Kerr et al., 2012a). Considering that one sixth of PWE are diagnosed with medication refractory epilepsy (Privitera, 2011), improved methods to effectively identify PWN who do not benefit from AEDs effectively could reduce the morbidity and both the financial and social cost of treating epilepsy.

Improved diagnostic tools could also help PWE. The difficulty in ruling out non-epileptic etiologies speaks to the challenge of adequately localizing and characterizing each patient’s epileptic etiology. The major seizure type discriminations are focal vs. generalized; partial vs. complex; and lesional vs. non-lesional. Each of these key discriminations leads patients down a different treatment path. When medication or other novel treatments like the vagus nerve stimulator fails, as they frequently do, the patient is left to consider resective neurosurgery. Recent reports have shown that surgery is most effective earlier in the course of disease (Engel et al., 2012). Improved diagnostic tools could more quickly and effectively diagnose patients with epileptic seizures and therefore speed the progression toward considering the surgical option.

Ultimately, our goal is to establish a general, automated computer-aided diagnostic (CAD) tool that effectively combines clinical information, manual interpretation of EEG and imaging technologies as well as automated analysis of interictal FDG-PET (iPET), EEG, structural MRI (sMRI), and diffusion MRI for all subtypes of epilepsy and NES. To accomplish this, we first must develop effective CAD tools that harness the information from each modality for a limited set of epileptic localizations. We have begun already to address automated analysis of interictal EEG for a wide variety of epilepsy subtypes (Kerr et al., 2012a). Others have described effective CAD tools that diagnose and lateralize temporal lobe epilepsy (TLE) using structural and diffusion MRI (Farid et al., 2012; Focke et al., 2012; Keihaninejad et al., 2012).

The clinical, metabolic, and structural differences between left and right TLE can be subtle. Some theories suggest that TLE is inherently a bilateral disease. Potentially, due to the strong functional link between the hippocampi, the only clinical difference is that in the aura of patient with left TLE (LTLE) more frequently includes language dysfunction. Over time, patients with LTLE more commonly develop verbal memory deficits, compared to non-verbal memory deficits in right TLE (RTLE) (Delaney et al., 1980; Kim et al., 2003). This functional connection between the hippocampi may also lead some patients to be falsely-lateralized using scalp EEG because a small seizure onset zone (SOZ) in one hippocampus can induce larger scale ictal activity in the contralateral hippocampus with very little time delay. This can lead neurologists to falsely conclude that the SOZ is either bilateral or in the contralateral hippocampus. Structural and metabolic imaging can reduce these errors by demonstrating that that one temporal lobe is asymmetrically affected, as shown by the previous CAD tools that lateralize TLE (Farid et al., 2012; Focke et al., 2012; Keihaninejad et al., 2012). Studies of the functional connectivity of these epileptic networks, however, conclude that there are very few, if any, differences between the two lateralizations (Zhang et al., 2010; Liao et al., 2011; Morgan et al., 2011, 2012; Pittau et al., 2012; McCormick et al., 2013). Recently, Pereira et al. (2010) suggested that more patterns of functional connectivity change in LTLE compared to RTLE. However, after patients suffer from intractable seizures for 10 or more years, the intrahemispheric hippocampal connectivity linearly increases with the duration of disease, suggesting that over time lateralized disease may become bilateral disease (Morgan et al., 2011). Because patients with bilateral hippocampal disease are no longer considered surgical candidates, improved methods to distinguish left and right TLE early in the course of disease are needed.

In this manuscript, we discuss the development of an automated CAD tool to diagnose, and lateralize, TLE using iPET. We also begin to address how to combine our CAD tool with manual analysis (MA) and incorporate it into clinical practice. Using a mutual information-based feature selection technique, we examine how our methods reveal more about the distributed metabolic abnormalities that are associated with the different anatomical locations of the epileptogenic focus.

The realistic goal of CAD tools is to complement, not to replace, expert analysis. Therefore, we focus on how clinical information and expert analysis can work synergistically with our automated technology. To summarize the major clinical differences, patients with NES are characteristically females in the third decade of life with psychiatric co-morbidities (Sahaya et al., 2011). PWE, however, also have significant psychiatric co-morbidities including potentially reduced financial and social independence due to the suspension of their driver’s and, frequently, professional license. Particularly in adult onset epilepsy, age-associated changes in metabolism may confound the interpretation of iPET, possibly leading to an increased diagnostic uncertainty. It is well established that 80–90% of medication refractory epilepsy is “PET positive” (Salamon et al., 2008; Lee and Salamon, 2009).The rate of PET positivity in NES has not been studied extensively, therefore the true positive predictive value of iPET is unclear. Although these differences in clinical presentation are salient, their quantitative effect on diagnostic probabilities is unknown. Therefore, we also examined how simple clinical information and expert manual interpretation can be incorporated into our quantitative CAD tool.

The standard of care for the pre-surgical assessment for epilepsy is the manual correlation of iPET with numerous other diagnostic modalities. The goal of this assessment is to simultaneously verify the diagnosis of epilepsy, characterize the seizure etiology, and identify the location and extent of the SOZ. Expert radiologists and neurologists can detect metabolic asymmetries indicative of the epileptogenic focus or foci (Person et al., 2010). The exact threshold at which asymmetric metabolism is attributed to pathologic change or seen as a variant of normal is part of the art of neuroradiology (Benbadis et al., 2000; Reuber et al., 2002). Once non-epileptic etiologies have been ruled out, our previous work demonstrated that the quantitative degree of metabolic asymmetry is correlated with surgical outcome (Lin et al., 2007). Surgical outcome is improved further when iPET is co-registered to sMRI because of improved characterization of the focus or foci (Chandra et al., 2006; Rastogi et al., 2008; Salamon et al., 2008; Lee and Salamon, 2009). These hypometabolic lesions are thought to be secondary to increased inhibitory neuron cell death, gliosis, and abnormal functional connectivity resulting in altered functional metabolism.

The size of the hypometabolic lesion tends to be larger than the SOZ, potentially due to functional changes in nearby tissue secondary to the presence of the epileptogenic lesion (Juhasz et al., 1999; Matheja et al., 2001; Henry and Roman, 2011). Such reports are major limitations to the wide implementation of iPET in epilepsy practices (Barrington et al., 1998; So et al., 2000; Henry et al., 2011). In addition to the limitation of counting statistics, that forces the quantitative radioactivity intensity of iPET to be less certain in hypometabolic lesions (Kerr and Lau, 2012), the biological hypothesis is that the epileptogenic abnormality induces metabolic abnormality at the SOZ and also at closely associated and/or functionally connected regions (Henry et al., 1990, 1993; Sperling et al., 1990; Sadzot et al., 1992; Arnold et al., 1996; Dlugos et al., 1999; Bouilleret et al., 2002; Rusu et al., 2005; Nelissen et al., 2006; Takaya et al., 2006; Lee et al., 2009). The epileptogenic lesion commonly is larger and more diffuse in left TLE then right TLE, potentially because of the high degree of functional connectivity between specialized foci within the left temporal lobe associated with language and other functions (Toga and Thompson, 2003; Barrick et al., 2005; Iturria-Medina et al., 2011; Haneef et al., 2012; Kucyi et al., 2012). These insights parallel the trend in dementia that atrophy starts focally then spreads more quickly to functionally connected regions (Zhou et al., 2012). The limited sensitivity of iPET unaligned with sMRI to characterize extratemporal lesions may be partly due to the insufficient description of the local functional network of each extratemporal focus and thereby reduced detection of a characteristic pattern of metabolic abnormalities associated with each focus. In general, an improved insight into the clinical interpretation and value of metabolic abnormalities outside the SOZ is needed. To overcome this limitation, the iPET analysis is used in combination with other diagnostic modalities determine which tissue to resect.

Clinical description, EEG, MRI, and FDG-PET each describe separate facets of the pathophysiological etiology, and therefore all play critical roles in the diagnosis of epilepsy, and in the identification of the epileptogenic lesion (Struck et al., 2011). Each modality, however, also has unique limitations. EEG provides an in-depth description of the seizures and interictal epileptiform spikes. These seizures and spikes, however, are rare events: only 50% of PWE exhibit diagnostic interictal epileptiform spikes and/or seizure activity during the first outpatient scalp EEG (Gilbert et al., 2003). The characteristic signs of epilepsy in structural and diffusion MRI may not be measurable until years after the first seizure because these methods require the detection of atrophic tissue and/or subtle regions of cortical dysplasia (Swartz et al., 1992; Reutens et al., 1996; Van Paesschen et al., 1998; Liu et al., 2002; Jung da and Lee, 2010; Bernasconi et al., 2011; Schmidt and Pohlmann-Eden, 2011; Dabbs et al., 2012). MA uses the contralateral structure to assess if atrophy is present but a certain degree of asymmetry is expected (Farid et al., 2012; Keihaninejad et al., 2012). It takes years of specific experience in manually analyzing sMRIs from PWE to reliably discriminate between normal variation and pathologic changes. Once these relatively large-scale changes in neural structure have occurred, it is less likely that both invasive and non-invasive treatments will be effective (Engel et al., 2012). iPET can localize the epileptogenic lesion without observing rare events and, potentially, before changes are detectible on sMRI and/or diffusion tensor imaging (DTI) (Theodore et al., 1990; Ryvlin et al., 1991; Swartz et al., 1992; Gaillard et al., 1995; Debets et al., 1997; Knowlton et al., 1997, 2008; Blum et al., 1998; Drzezga et al., 1999; Benedek et al., 2004; Carne et al., 2004; Chandra et al., 2006; Yun et al., 2006; Uijl et al., 2007; Willmann et al., 2007; Rastogi et al., 2008; Salamon et al., 2008; Duncan, 2009; Lee and Salamon, 2009; Lerner et al., 2009; Liew et al., 2009; Brodbeck et al., 2010; Chinchure et al., 2010; Kim et al., 2011; Jupp et al., 2012). As discussed above, the presence of metabolic abnormalities outside the SOZ, however, complicates the effective localization of the SOZ using iPET alone (Henry and Roman, 2011). An improved description of these induced changes outside the SOZ may help spare healthy tissue from resective surgery. Given the recent report that resective neurosurgery for epilepsy is more effective earlier in disease (Engel et al., 2012); we believe that iPET may play a critical role in characterizing patients with unremarkable MRIs and inconclusive EEGs earlier in the course of their disease.

## Materials and Methods

### Patient data

All of the 105 patients that were included in our analysis were admitted to the University of California, Los Angeles (UCLA) Seizure Disorder Center’s video-EEG Epilepsy Monitoring Unit (EMU) between 2005 and 2012. Each patient’s diagnosis was based on a consensus panel review of their clinical history, physical and neurological exam, neuropsychiatric testing, video-EEG, iPET, ictal FDG-PET, structural and diffusion MRI, and/or CT scan. This multimodal assessment is the gold standard for epilepsy diagnosis and localization of the epileptic focus (Cragar et al., 2002; LaFrance and Devinsky, 2004). The patients included in this analysis were chosen because they had an FDG-PET after 2005; had no history of penetrative neurotrauma, including neurosurgery; were determined by consensus diagnosis to have a single, lateralized epileptogenic focus; and had no suspicion of mixed non-epileptic and epileptic seizure disorder. These patients were diagnosed either with LTLE (*n* = 39), right TLE (RTLE, *n* = 34), or NES (NES, *n* = 32). PET images were determined to be interictal by clinical findings and concurrent scalp EEG.

PET and MRI images were acquired according to the best clinical practices at the time of acquisition. PET/CT studies were acquired using a Siemens Biograph scanner. After a minimum fasting period of 6 h, patients received 0.14 mCi/kg of 18F-FDG-PET intravenously. During the ensuing 40 min uptake period with concomitant EEG monitoring to confirm interictal status, the patients waited in a quiet, dimply lit room with their eyes open. PET images were reconstructed with an iterative algorithm (OSEM: 2 iterations, 8 subsets). CT images were reconstructed using filtered back projection at 3.4 mm axial intervals to match the slice separation of the PET data, and used for attenuation correction.

### Computer-aided diagnostic tool training and validation

Automated analysis of the iPET records was performed in four stages. (1) First, each image was screened for gross structural and/or metabolic abnormalities by S.T.N., N.M.R., and/or W.T.K. (*n* = 21). These excluded subjects are not reflected in the sample sizes quoted above. (2) NeuroQ (Syntermed, GA, USA) was used to segment each brain into 47 regions of interest (ROIs) and then to calculate the average radioactivity in each ROI, normalized by the whole brain radioactivity (Table A1 in Appendix). (3) The minimum redundancy-maximum relevancy (mRMR) toolbox for MATLAB (Mathworks, MA, USA) was used to generate a ranked list of the ROI metabolisms (features) within each training set that were maximally relevant to the diagnosis of epilepsy and minimally redundant with all higher ranked features (Ding and Peng, 2005; Peng et al., 2005). The representative number of features to exclude and quantal levels was selected based on our method discussed previously (Kerr et al., 2012a,b) (see below). In each of the training sets, the feature ranking was determined exclusive of the test patient’s data. We expect the ranked lists to be similar, but not identical, across training sets. For purely illustrative purposes, the full dataset was used to create the ranked list in Table 2. (4) Weka was used to implement leave-one-out cross-validation of a cost-sensitive Multilayer Perceptron (MLP) that was weighted to maximize balanced accuracy, defined by the mean of sensitivity and specificity (Bouckaert et al., 2010). Using this method, we examined our ability to diagnose either LTLE or RTLE from NES and assessed our ability to diagnose and lateralize disease simultaneously. For the remainder of this manuscript, the latter tool that discriminates LTLE vs. RTLE vs. NES is called the trinary classifier. Similarly, the binary CAD tools are referred to by the laterality of epilepsy that is being detected. The comparison to NES is not stated, but can be assumed. We then compared our CAD tool’s performance to the results of MA alone.

### Machine learning algorithmic details

The MLP was implemented with default parameters in Weka (Bouckaert et al., 2010). All input features were normalized to values between negative and positive 1. No limit was set on the number of hidden layers or nodes within each hidden layer. These parameters were optimized within each training set independently. The learning rate and momentum were set to 0.3 and 0.2, respectively. Five hundred epochs were used for training. During training, models with more than 20 consecutive errors were excluded. The trinary classifier was created by decomposing the three class problem into three 1-against-1 problems that were combined using majority voting. No three-way ties occurred during training or testing.

Balanced accuracy was optimized using a cost-sensitive classifier in which a false positive was given a cost of *n*_+_ and a false negative was given the cost of n_−_, where n_+_ and n_−_ represent the number of PWE and NES in the full sample, respectively. In the trinary classifier, the cost was set as the sum of the number of patients in the other two diagnostic classes.

Cyclical leave-one-out cross-validation (CL1OCV) was used to assess the performance of the MLP. In this paradigm, all but one patient was used to determine the features selected and train the algorithm. The single remaining patient is tested using the model built upon the other patients. The identity of the test patient is permuted until all patients have been the test case once and only once.

To determine the number and identity of the input features, the mRMR algorithm requires the number of input features, *F*, and quantal levels, *Q*, be set *a priori*. For the calculation of mutual information, the features were smoothed into *Q* quantal bins akin to the bins in a histogram. Classification, however, utilizes unsmoothed features. The choice of input features smoothed into quantal levels was determined to be most representative of the performance of the algorithm across a wide variety of choices of *F* and *Q* (Kerr et al., 2012b). This choice was made by selecting a point within a region of *F-Q* parameter space that performed significantly better than the naïve classifier with 95% confidence based on random field theory correction where the spatial smoothness is estimated directly from the data (for more details, see Worsley et al., 1992, 2004; Chauvin et al., 2005). The naïve classifier classifies all test exemplars as the most common class in the training set. Under the CL1OCV procedure, these input features were determined independently for each of the training samples. The illustrated rank order of features was calculated based on the full dataset, and does not necessarily match the rank list of any individual training sample.

When clinical information was incorporated into the algorithm, the same methodology was applied as above, except that all exemplars with missing data were excluded from analysis. In these additional analyses, we did not re-sample the parameter space of *F* and *Q*. We simply used the selections determined in the previous analysis.

### Manual analysis of PET and MRI records

Manual analyses of the iPET and sMRI records were performed based on the review of clinical records primarily written by Dr. Noriko Salamon. Dr. Salamon has 10 years of experience in the pre-surgical assessment of epilepsy using FDG-PET and MRI. All manual interpretation was conducted for the clinical assessment of each patient when it occurred, prior to the CAD tool development. Therefore, Dr. Salamon was blinded to the automated results. Due to the unclear relationship between structural and metabolic abnormalities, asymmetries, and epilepsy, all abnormal results were interpreted to be consistent with some form of epilepsy. Not all patients had sMRI (*n* = 6) and iPET (*n* = 1) reports available; therefore all analysis regarding MA of neuroimaging includes only patients with available records. These patients had raw iPET data available; they therefore were included in the automated analysis.

### Combination of clinical information with computer-aided diagnostic information

To examine the combined power of clinical knowledge, MA and our automated analysis, we assessed the linear correlation of detecting epilepsy with CAD compared to MA, and also incorporated clinical information and MA into our algorithm in two ways. First, the clinical literature suggests that patients with NES are more likely to be female, begin having seizures in the third decade of life, have a decreased duration of disease and have increased seizure frequency (Table 1). Although we did not see a significant difference seizure frequency within our dataset, we included this features to better match clinical practice. These clinical features were then added to the input and leave-one-out cross-validation was repeated. Secondly, to explore how our computational methods can complement clinical wisdom, we included the results of MA of the iPET and sMRI as two additional input features and re-evaluated CAD performance. For the trinary classifier only, we split each of the features describing the iPET and sMRI MA to indicate if a left and/or right sided abnormality was reported.

**Table 1:** **Clinical information and results of manual analysis**.

		NES	LTLE	RTLE
Age	Mean ± SD	37 ± 14*	38 ± 12	36 ± 13^¶^
	Min-Max (Median)	16–76 (38)	18–54 (40)	17–67 (35)
	*N*	32	39	34
Sex	% Female ± SE	78.1 ± 7.3*^§^	53.8 ± 8.0	35.3 ± 8.2
Duration of disease	Mean ± SD	12 ± 12*^§^	22 ± 15	20 ± 13
	Min-Max (Median)	10 d–40 y (7)	6 m–53 y (21)	2 y–48 y (19)
Seizure frequency	Mean ± SD	3.2/d ± 5.9/d	1.2/d ± 2.4/d	1.5/w ± 1.7/w
	Min-Max (Median)	0.3/m–25/d (3/d)	0.2/m–11/d (1/w)	0.1/m–1/d (0.8/w)
iPET manual	% Positive ± SE	18.8 ± 6.9*^§^	76.9 ± 6.7	87.9 ± 5.7
	*N*	32	39	33
sMRI Manual	% Positive ± SE	34.5 ± 8.8*^§^	73.7 ± 7.1	87.5 ± 5.8
	*N*	29	38	32

This table reflects the clinical information known before the application of the CAD tool. All times are listed in years (y) unless otherwise specified by days (d), weeks (w), or months (m). Manual analysis of all patients’ iPET and sMRI were not done, therefore we list the number with available manual results. *, ^§^, or ^¶^ indicate that the value for NES vs. LTLE, NES vs. RTLE, or LTLE vs. RTLE, respectively, is statistically significant from both the LTLE and RTLE groups with at least 95% confidence using a two-sample *z*-test of proportions or Mann–Whitney *U* test, where appropriate. No other differences are statistically significant (*p* > 0.10).

To assess the applicability of our CAD as a *separate* modality that could be considered as part of the clinical assessment of epilepsy, we calculated the likelihood ratios (LRs) of each of the combinations of our CAD with MA of iPET and/or sMRI. This was done only for the binary classifiers, because LRs have a clear formulation only for binary outcomes. The likelihood ratio is defined by the likelihood that a patient with a certain combination of diagnostic outcomes has epilepsy, divided by the likelihood that the same patient has NES. Intuitively, a likelihood ratio of two implies that the patient is twice as likely to have epilepsy. The 95% confidence intervals of chance were calculated using exact binomial intervals by considering the likelihood ratio of a classifier that diagnosed patients according to their prior likelihood alone, conditioned upon the assumption that the same total number of patients would have the diagnostic outcome of interest. For example, 39 of 71 patients had LTLE when we discriminated between LTLE and NES, therefore the median LR is 1.2. Thirty-five patients from the NES vs. LTLE group had negative MA of their iPET. Therefore, we use a binomial distribution with 35 trials and success probability of 39 over 71 to yield a 95% confidence interval of 0.94–3.38.

## Results

**Figure 1:**
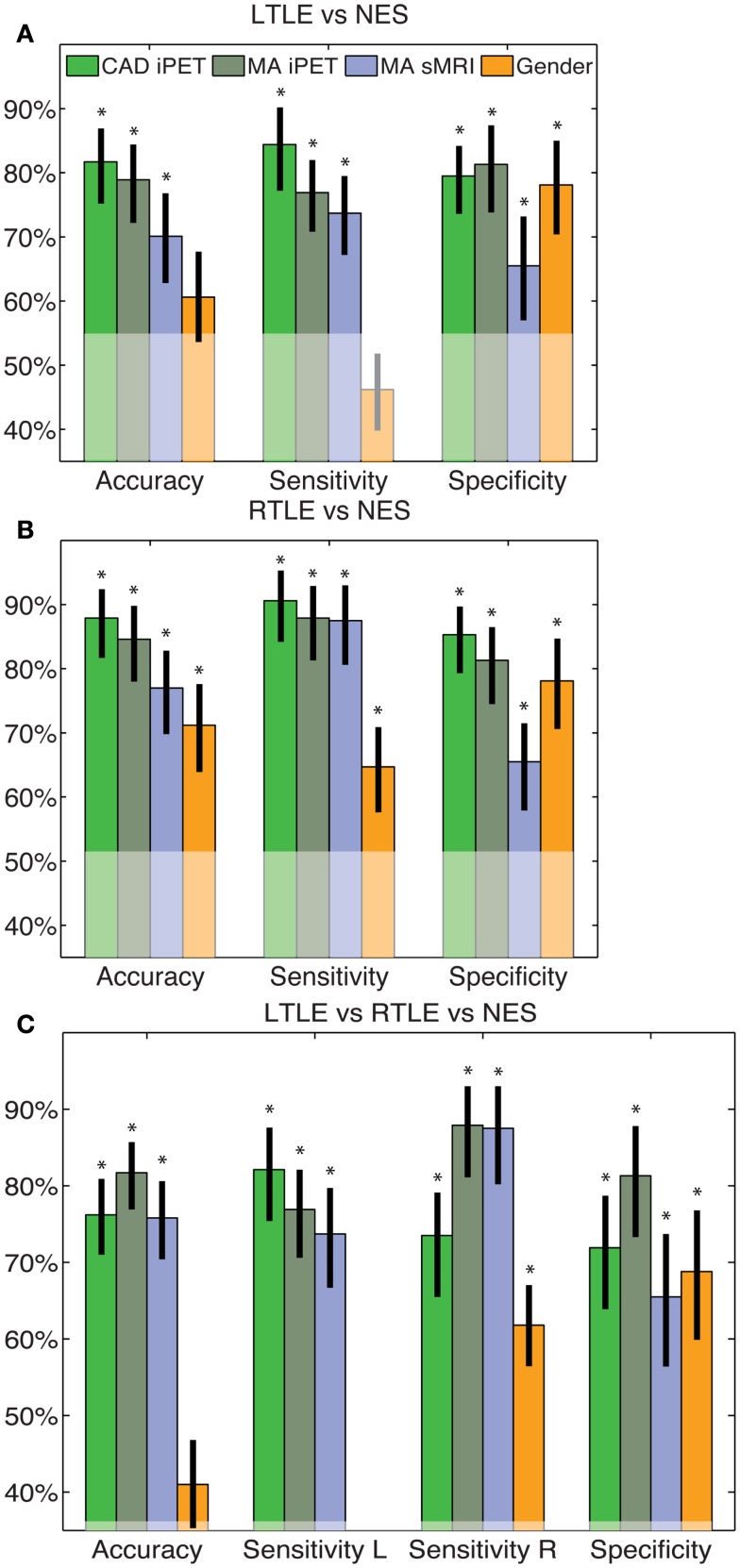
**CAD tool performance matches manual analysis**. These figures indicate the accuracy, sensitivity and specificity of the LTLE **(A)**, RTLE **(B)** and trinary **(C)** classifiers. The performance of our CAD tools matched that of MA and was superior to just using gender alone. The error bars indicate standard error of the mean performance for each measure. The translucent region indicates the performance of a naïve classifier. *Indicates significant differences from the naïve classifier with a confidence level of 95% or more.

All of our results are compared to the gold standard diagnosis from the consensus panel. The clinical trial statistics of each of our automated diagnostic tool matched, but were not redundant with, expert MA of both interictal PET and sMRI (Figure 1). All intervals reflect 95% confidence intervals and all *p*-values correspond to differences from anaïve classifier. The binary CAD tool for RTLE had accuracy of 88% (69–90%), compared to the accuracy of MA of iPET [85% (72–92%)] and sMRI [77% (63–85)]. The binary tool for LTLE had accuracy of 83% (69–90%), compared to the accuracy of MA of iPET [79% (66–88%)] and sMRI [70% (56–81%)]. The pattern in sensitivities, specificities, and odds ratios all parallel this trend where our automated diagnostic tools are non-statistically superior to MA oriPET, which, in turn, are non-statistically superior to MA of sMRI (Figure 1). The accuracy of our trinary CAD tool that simultaneously diagnoses epilepsy and lateralize disease was 76% (66–84%), where 89% (77–96%) of patients correctly identified with epilepsy were also lateralized correctly. MA to diagnose and lateralize was 78% (69–86%) accurate with 89% (76–94%) correctly lateralized using iPET and 71% (61–80%) accurate with 91% (78–97%) correctly lateralized using sMRI.

The rank order of the features used in our algorithm parallel the clinical observation that the epileptogenic networks in LTLE are broader than in RTLE. The LTLE vs. NES classifier achieved its performance by utilizing trends across almost the entire brain by including 42 of the 47 features in the final algorithm. In contrast, the RTLE vs. NES classifier only needed to measure the metabolism in six regions – bilateral temporal cortex and two associated regions of cortex – to achieve its impressive performance (Table 2). As expected, the trinary classifier utilized an intermediate number of features to achieve its accuracy (30 of 47). The rank list of these features matches the biological intuition based on knowledge about the potential connectivity of epileptogenic networks (Table 2).

**Figure 2:**
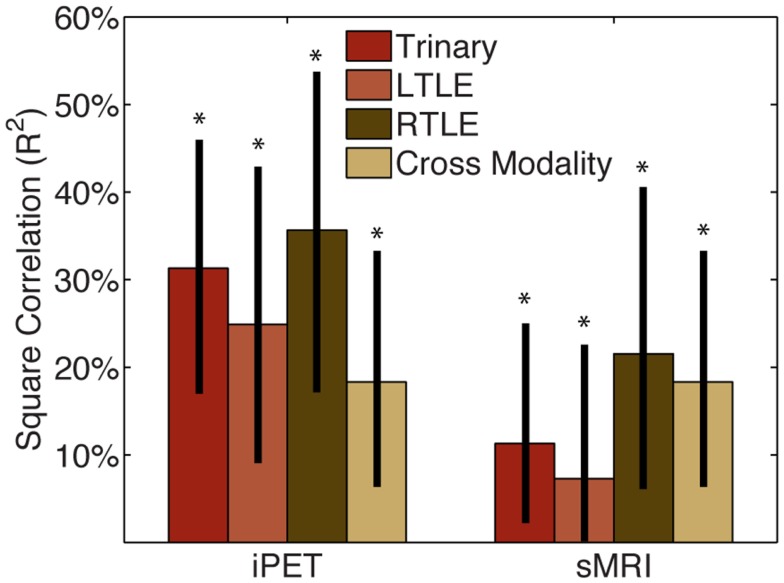
**CAD tool is not redundant with manual analysis**. The squared correlation of our CAD tools’ results with those of MA of the iPET or sMRI from the same patients was below 50%. This indicates that while some information is shared, the majority of information provided by our CAD tools is not captured by MA. The correlation between MA of iPET and sMRI is similar in magnitude to the correlation of CAD with MA, therefore the CAD could potentially be seen as similar to another informative modality. *Indicates significant differences of the correlation from zero with a confidence level of 95% or more.

We then considered how this CAD information could be used in combination with clinical information or expert analysis. The squared correlation of our CAD tool with manually interpreted iPET was 0.25 (0.09–0.43), 0.32 (0.17–0.54), and 0.34 (0.17–0.46) for the LTLE, RTLE, and trinary classifiers, respectively (Figure 2). The squared correlation of our tool with manually interpreted sMRI was 0.07 (0.001–0.23), 0.21 (0.06–0.40), and 0.11 (0.02–0.25) for the LTLE, RTLE, and trinary classifiers respectively. For comparison, the squared correlation between manually interpreted iPET and sMRI was 0.17 (0.06–0.33).

**Table 2:** **Ranked list of contributing metabolic ROIs**.

	Region of interest		
mRMR rank	LTLE vs. NES	RTLE vs. NES	Trinary
1	Midbrain	R ila temporal C	R ila temporal C
2	L ilp temporal C	R ilp temporal C	L ilp temporal C
3	R ilp temporal C	L sensorimotor C	L sensorimotor C
4	L associative visual C	L sl temporal C	R ilp temproal C
5	L Broca’s Region	R thalamus	R sl temporal C
6	L s frontal C	R i frontal C	R pm temporal C

*This table illustrates the top six informative and non-redundant regions of interest (ROIs) that may contribute to each of the CAD tools, as determined by the minimum redundancy-maximum relevancy criteria (mRMR; Ding and Peng, 2005; Peng et al., 2005). The illustrated rank order of features was calculated based on the full dataset and does not necessarily match the rank list of any individual training sample. The leading L or R indicates left or right. The lowercase letters indicate inferior (i), lateral (l), median (m), anterior (a), and posterior (p). The lagging C signifies cortex. Note that the LTLE vs. NES and trinary classifiers include information from 42 and 30 ROIs, respectively. To better understand the benefit of mRMR, this list can be directly compared to the list of ROIs ranked by *t*-statistics in Table A1 in Appendix*.

**Figure 3:**
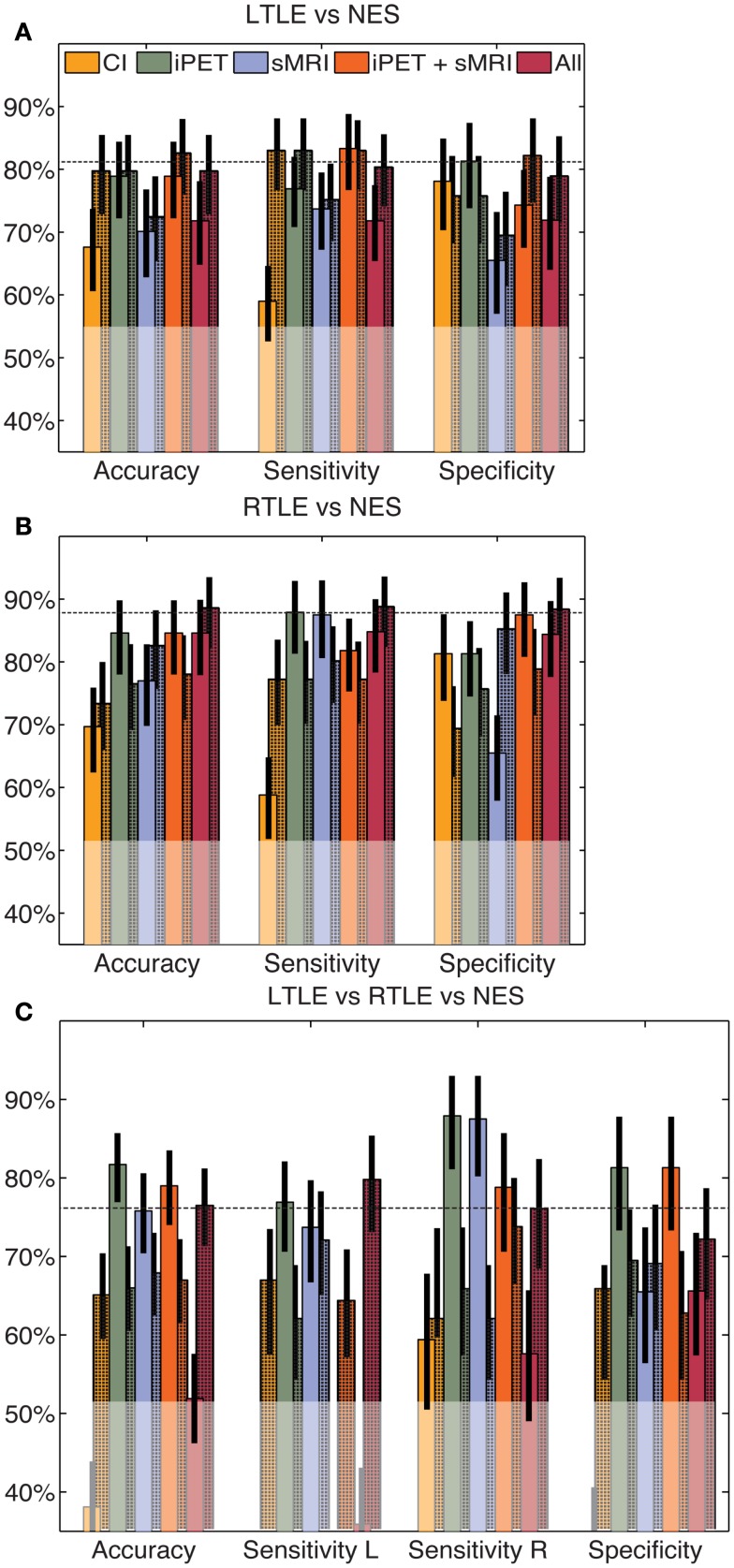
**Automated combination of clinical information with automated analysis of iPET images**. The automated combination of clinical information and/or MA with our analysis produced no significant change in performance for the LTLE **(A)**, RTLE **(B)** or trinary **(C)** classifiers, relative to the CAD operating on automated values alone. The unshaded bars indicate the performance of similarly constructed CAD tools using clinical information or the results of MA alone. The shaded bars indicate the modified performance when information from NeuroQ is added. The horizontal line indicates the mean accuracy of each CAD tool without clinical information. The translucent region indicates the performance of a naïve classifier.

When the same automated analysis was used to combine clinical findings with our iPET data, performance did not change significantly. After the four clinical factors were added to the input of our tools, the accuracy changed to 79% (66–88%), 68% (56–79%), and 64% (54–73%) for the LTLE, RTLE, and trinary classifiers, respectively (Figure 3). These accuracies do not substantively change when only sex and duration of disease were considered (results not shown). Adding the results of MA of both iPET and sMRI to our iPET data changed the accuracy to 82% (73–91%), 77% (67–88%), and 68% (59–77%) for the LTLE, RTLE, and trinary classifiers, respectively. When all information sources contribute to the algorithm, the accuracy changed to 77% (68–88%), 74% (64–85%), and 76% (68–84%) for the LTLE, RTLE, and trinary classifiers, respectively.

**Figure 4:**
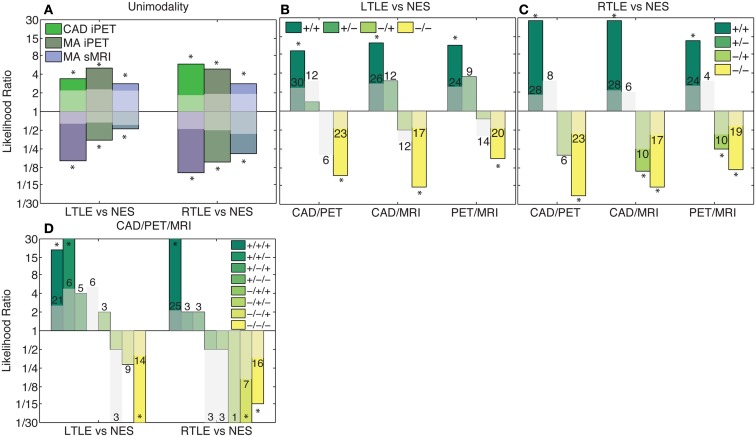
**Combination of clinical information and CAD results using likelihoods**. Columns in this log plot above 1 indicate that the seizures are more likely to be epileptic whereas the columns below 1 indicate a non-epileptic etiology is more probable. **(A)** Illustrates the positive and negative likelihood ratio of each analysis method considered individually. **(B,C)** Illustrate the likelihood ratios of each possible outcome when two analysis methods are combined. **(D)** Indicates the likelihood ratios of each possible outcome when all analysis methods are combined. If all modalities agree, the likelihood non-significantly increases with the addition of each modality. However, if there is disagreement, the likelihood ratio is generally not significantly different from chance. The translucent bars indicate the 95% confidence interval for chance with the relevant sign (see Materials and Methods).The numbers above the translucent bars indicate the total number of patients with each outcome. The bars that go off the scale of the graph diverge toward zero or infinity because no patients of a certain class had that outcome. *Indicates significant differences of the correlation from zero with a confidence level of 95% or more.

We combined the results of MA were combined with our CAD tool manually using LRs. After doing so, the likelihood was generally only significant if all considered modalities agreed. Viewed alone, MA and our CAD increased the likelihood of the predicted outcome between two and ninefold (*p* < 0.02; Figure 4A). When two analysis streams were combined, if both analyses agreed, the likelihood of the predicted outcome was increased between 8- and 27-fold (*p* < 3 × 10^−4^; Figures 4B,C). If all three analyses agreed, the likelihood of the predicted outcome increased more than 15-fold (*p* < 1.3 × 10^−5^; Figure 4D). However, in most cases, if there was any disagreement, the likelihood did not change significantly, most probably due to the small numbers of patients with each potential outcome. There are two key exceptions: (1) Given iPET results indicating NES over RTLE using either MA or CAD, the sMRI could be largely ignored (*p* < 1.1 × 10^−2^). (2) If both MA and CAD of iPET agreed that a patient suffered from LTLE and not NES, the sMRI results could be similarly ignored (*p* < 3.3 × 10^−2^).

## Discussion

These results demonstrate how our CAD tool has the potential for clinically application, while also confirming and elucidating the distributed effects of epilepsy on the entire brain. Our CAD tool’s diagnostic performance of TLE matches, but is not redundant with, expert MA of iPET and sMRI. When considered in the context of recent reports of CAD tools for epilepsy based on sMRI and interictal EEG data (Farid et al., 2012; Focke et al., 2012; Keihaninejad et al., 2012; Kerr et al., 2012a), CAD is proving especially applicable to epilepsy. Further, if more work confirms the hypothesis that metabolic changes in iPET are observable before the structural changes in sMRI, our iPET tool may have better clinical utility than these existing sMRI tools. In contrast to MA, this and other CAD tools can be quickly and efficiently applied by minimally trained technicians, emergency physicians, and primary care providers as preliminary analysis of the iPET images (van Ginneken et al., 2011; Kerr et al., 2012c). The performance of MA can vary with experience and fatigue of the observer; automated tools are consistent over time. Upon further validation, these CAD results could also be incorporated into the consensus diagnoses with minimal cost if iPET already has been obtained.

### Clinical impact

Our CAD tools could provide valuable clinical information that may help readily identify which treatments may be effective in patients who present with uncharacterized, and/or medication refractory seizures (Kerr et al., 2012a,c). In particular, 15 of our 105 patients were admitted twice to achieve definitive characterization or localization of their seizures. The appropriate binary classifier correctly diagnosed 12 (80%) of these challenging patients. This valuable information might reduce the need for multiple video-EEG admissions. Additionally, 28% (9/32) of our PWN were admitted for improved characterization of their previously-diagnosed “epilepsy,” and 16% (12/73) of our PWE were admitted for the differential diagnosis of epilepsy, indicating that non-epileptic etiologies were not ruled out sufficiently. The trinary CAD effectively diagnosed 67% (14) of these particularly challenging patients. Despite this impressive performance, the ultimate goal of CAD, however, is to complement – not replace – MA.

### Combination of automated analysis with clinical wisdom

Our finding that performance almost uniformly, but non-statistically, decreased when the automated algorithm incorporated clinical information indicates that automated analysis cannot and should not replace manual interpretation across information modalities. We suspect that this performance decreased due to ineffective modeling of the contribution of the clinical information and over-fitting. The statistical distribution of the clinical factors was very different from the metabolic data therefore the same model likely cannot effectively utilize both modalities. The efficient incorporation of multimodality information into machine learning is an active area of theoretical research, and well-validated methods are not yet available. Now that CAD tools using interictal EEG (Kerr et al., 2012a), sMRI (Farid et al., 2012; Focke et al., 2012; Keihaninejad et al., 2012), and iPET have been published, we believe it will be extremely exciting to assess how these various tools can be combined.

We expected that the best performance would be achieved when our CAD is used synergistically with MA. The low correlations between the CAD results and MA suggest that our CAD tool provides information that is not evident on visual inspection. These results emphasize that PET is not redundant with MRI (Henry et al., 1999). Physicians could learn to view CAD as analogous to another imaging modality that provides valuable, but not perfectly diagnostic, clinical insight. This synergistic application of computer-aided diagnosis after manual interpretation already has proven beneficial in the detection of lung nodules by the FDA and is an active area of translational research (Kerr et al., 2012c; Wang et al., 2012). The key differences between MA and automated analysis are the ability to entirely ignore certain pieces of data, and to rule that the results are inconclusive.

The results summarized above, and the LRs for each analysis stream individually, show that both MA and CAD are useful clinically. If the analysis streams agree, the diagnostic certainty increases substantially, but at a cost: as more analyses are added, more patients have inconclusive results because the analyses did not agree, and the LRs are not significant. Even though our sample size is large compared to other studies of this type, there were not enough patients in our dataset with each diagnostic outcome to explain the clinical implication of disagreeing analyses adequately. This matter of inconclusive results is a common challenge faced in clinical practice. Physicians struggle regularly with those types of decisions. When MA of iPET and sMRI are combined, they need to agree to yield meaningful results. However, our analysis shows that in some specific cases, if both the MA and CAD of iPET agree, the sMRI is not needed. This parallels the finding we suggested above: iPET may be more clinically useful than sMRI to diagnose and lateralized epilepsy. The hypometabolic abnormality may be present earlier in disease (Theodore et al., 1990; Ryvlin et al., 1991; Swartz et al., 1992; Gaillard et al., 1995; Debets et al., 1997; Knowlton et al., 1997, 2008; Blum et al., 1998; Drzezga et al., 1999; Benedek et al., 2004; Carne et al., 2004; Chandra et al., 2006; Yun et al., 2006; Uijl et al., 2007; Willmann et al., 2007; Rastogi et al., 2008; Salamon et al., 2008; Duncan, 2009; Lee and Salamon, 2009; Lerner et al., 2009; Liew et al., 2009; Brodbeck et al., 2010; Chinchure et al., 2010; Kim et al., 2011; Jupp et al., 2012), and it may provide slightly more accurate disease characterization, as seen in our dataset. In settings where the PET scanner is not combined with the MRI scanner, and/or when the cost of imaging is a limiting factor (both common occurrences) the effective application of our CAD could result in substantial cost savings.

### Pathophysiological insights

Our methods also reveal a potential difference in the pathophysiology of left vs. right TLE. This may help explain why CAD tools perform slightly better when diagnosing RTLE compared to LTLE (Farid et al., 2012; Focke et al., 2012; Keihaninejad et al., 2012). The finding that mostly bilateral temporal ROIs, the right inferior frontal cortex and left sensorimotor cortex provide non-redundant diagnostic information for RTLE is consistent with the clinical wisdom that the epileptogenic network in RTLE is more focal than in LTLE. The inclusion of temporal regions echoes the conventional wisdom that focal hypometabolism and asymmetry reflect characteristic changes due to epilepsy. This suggests that conservative resection of the temporal lobe may result in increased rates of seizure freedom in RTLE compared to LTLE due to complete resection of the SOZ. Further, seizures that originate in the left temporal lobe may secondarily generalize more frequently in LTLE. These differences have not yet been studied clinically.

The trends in the extratemporal regions included in the algorithms suggest that the primary lesion may induce metabolic changes in functionally or anatomically associated regions. This is substantiated further by the finding that almost all regions of the brain provide informative diagnostic information in LTLE. This in turn mirrors the increased stereotypic connectivity of the left temporal lobe. Even though the interconnectivity of the right hemisphere is higher than the left hemisphere, the left hemisphere has strong connections between specialized foci (Barrick et al., 2005; Iturria-Medina et al., 2011; Kucyi et al., 2012). We hypothesize that the SOZ may induce abnormal metabolism along these strong, stereotyped connections. This change cannot be attributed to language specifically in our dataset because we did not identify the laterality of language dominance in our patients. Compared to our *t*-statistics ranking, it may seem surprising that the metabolism of the midbrain was ranked first by mRMR for LTLE vs. NES. This rank may indicate a non-linear change in the metabolism within the dorsal midbrain anticonvulsant zone, which has itself been identified in animals to be part of the network that modulates seizure threshold (Shehab et al., 1995). The exact relationship between epilepsy and midbrain metabolism is unclear, however. The lack of distributed atrophy in LTLE measured by sMRI suggests that these changes are not associated with distributed cell death or gliosis (Farid et al., 2012; Focke et al., 2012; Keihaninejad et al., 2012). Instead, we hypothesize that this change instead reflects abnormal metabolism in these regions due to altered neural connectivity and/or activity secondary to the epileptogenic lesion. This is supported by the finding that LTLE was associated with more changes in functional connectivity than RTLE was (Pereira et al., 2010). This also explains why we observed metabolic changes in the right thalamus in RTLE: recent work demonstrates that the connectivity of the right thalamus with the right hippocampus is reduced in RTLE (Morgan et al., 2012). The presence of such distributed changes also supports the finding that the size of the hypometabolic lesion visualized on PET may be larger than the SOZ (Juhasz et al., 1999; Matheja et al., 2001; Henry and Roman, 2011). It is particularly interesting to note that the extent of these distributed changes is underappreciated by *t*-statistics comparing LTLE to NES. This indicates that there is a complex, likely non-linear, relationship between the metabolism of the hypometabolic lesion and its associated tissue that may be better understood by mutual information.

The inclusion of the contralateral hippocampus in both of the binary classifiers lends itself to multiple interpretations that are all supported by biologically sound hypotheses. Firstly, a salient feature of LTLE or RLTE could be asymmetric metabolism, as suggested clinically; therefore the metabolism of the contralateral hippocampus was compared to the observed metabolism in the ipsilateral hippocampus. Alternatively, the interhemispheric connectivity between the hippocampi is high, therefore under our hypothesis that changes in metabolism spread according to functional connections, the metabolism in the contralateral hippocampus may be one of the first induced changes due to the epileptic lesion. Lastly, if LTLE and RTLE are inherently bilateral diseases then the metabolism in the contralateral hippocampus may also be abnormal. This also provides an explanation for why LTLE and RTLE were not perfectly distinguished.

In addition to diagnosing epilepsy, our algorithm lateralized disease efficiently with an accuracy of approximately 90% when epilepsy was diagnosed correctly. This impressive accuracy could be clinically useful for pre-surgical planning, when used in combination with other clinical and radiological information. Although our current sample size is too small to fully assess this potential fully, our results suggest that similar methodology could be applied to a larger dataset with more diverse and specific SOZ localizations to yield an objective and reliable tool to assist in pre-surgical SOZ localization. Our data suggest that this approach likely would identify and utilize distributed metabolic findings associated with each epileptic lesion to improve performance. Instead of blurring the boundary of the SOZ by detecting affected tissue outside the SOZ, the improved understanding of these distributed effects may lead to more refined characterization of this clinically vital SOZ. However, the spatial resolutions of our outcome classes were insufficient to assess the utility of this method directly to identify candidate lesions for resective surgery.

While our lateralization accuracy is exciting, there is also a potential clinical interpretation of the patients who were falsely-lateralized. Functional connectivity between the temporal lobes is particularly strong. In a minority of patients, this connectivity allows epileptogenic activity to spread quickly from the SOZ to the contralateral temporal lobe on EEG, resulting in the appearance of either bilateral or falsely-lateralized disease. Similarly to the distributed networks discussed above, this high degree of functional connectivity also may induce metabolic abnormalities in the contralateral temporal lobe that may be indistinguishable from the primary lesion. This hypothesis can be tested by comparing these falsely-lateralized patients to patients with bilateral TLE. This comparison requires a detailed methodological treatment of non-mutually exclusive classes in machine learning and therefore lies outside the scope of the current manuscript.

To characterize these and other pathophysiological insights, most studies utilize healthy neurologically normal controls. In contrast, we prefer the use of PWN as our control group. In brief, when constructing a control group, one aims to match the patients in the pathologic group in all aspects other than the pathology. In contrast to neurologically normal controls when compared to PWE, PWN’s have been exposed similarly to AEDs and other medications, have increased prevalence of TBI and some other risk factors for epilepsy (Sahaya et al., 2011), have regular and frequent meetings with health care providers, and have much more strict inclusion criteria. Lastly, and perhaps most importantly, physicians do not consider whether all of their patients have epilepsy; they assess only the patients with seizures. Therefore, in our opinion, the use of PWN as the control group is a benefit in of our study because it maximizes the clinical relevance of our results while simultaneously improving its statistical selectivity.

### Limitations and future directions

Because our retrospective dataset was collected as part of clinical care, our approach has a few important limitations. The accuracy of MA reported in our patients is worse than the rates quoted in previous literature (Rastogi et al., 2008; Salamon et al., 2008; Lee and Salamon, 2009). Given UCLA’s status as a tertiary referral center, the decrease in manual accuracy likely indicates that our patients had more heterogeneous etiologies and/or were more complex and difficult to diagnose than other centers. This suggests that our CAD tool may perform better on other datasets. Our iPETs and MRIs were collected on varying cameras with varying resolutions. This demonstrates the flexibility of our automated analysis using NeuroQ. The efficacy of the MA of older and limited resolution data may not be comparable to that of more current and higher resolution data. After establishing the efficacy of our method, we plan to both validate our tool prospectively on data from other centers, and to incorporate multi-center data into our algorithm to further improve its performance. Additionally, we only discuss the combination of CAD results with independently derived MA. Future work will examine the efficacy of CAD tools informed by MA and vice versa.

Critics of our approach might claim that the significant gender and age difference of the patients with NES compared to PWE may lead to our CAD simply detecting the age and/or gender of the patients. While we do not expect this to be the case for RTLE, the utilization of language areas by the LTLE classifier might reflect differences in gender, and not epileptogenic pathology. However, the performance of our CAD was significantly higher than when clinical information was used directly, therefore the algorithm utilized more information than just clinical data to achieve its strong performance. These significant differences in clinical factors largely mirror the observed differences in clinic; therefore our dataset better matches the population for which our CAD tool would be applied. The only notable exception is the significant age difference between LTLE and RTLE, which was unexpected. Due to the naturalistic nature of our data collection scheme, we did not correct for this difference. However, we note similarly to the NES group, the use of age alone was significantly worse than our tool and the addition of age to the iPET data to control for its effect did not significantly change performance.

Another key caveat to the direct clinical application of our tool to clinical practice is the fact that epilepsy is an extremely heterogeneous disease. The generalization of our method to bilateral TLE, extratemporal foci and multifocal epilepsy will be critical before it can be incorporated into clinical practice. In particular, even though NES mimic all types of seizures, it is uncommon for TLE to be mistaken for NES. Instead, it is more common that NES appear to have a focus in frontal cortex (LaFrance and Benbadis, 2011). Therefore, the literature suggests that the highest impact CAD tool would discriminate between frontal lobe epilepsy and NES and another, separate tool could be used to lateralize TLE. Based on our results above (see section Clinical Impact), we believe that our TLE-specific tool may be clinically applicable. For the first publication demonstrating the applicability of computer-aided diagnosis based on iPET data, we chose to focus on the diagnosis and lateralization of TLE, based, based on prior findings that the sensitivity of iPET is highest for TLE. Our future work then can address generalizing our methods to the other epilepsies, including bilateral TLE and frontal lobe epilepsy.

### Conclusion

Despite a few caveats, and upon further validation with data from other centers, our automated methods could provide unique information for the effective and efficient characterization of epilepsy, with the potential to decrease the fraction of patients with NES that are being treated (inappropriately) with AEDs, and to more quickly triage patients with medication refractory epilepsy toward surgical intervention. This may help achieve the ultimate goal: a global reduction in seizures (Engel et al., 2012).

## Conflict of Interest Statement

Daniel H. Silverman is a co-inventor of NeuroQ, which is licensed to the University of California by Syntermed. The other authors have no conflicts of interest to disclose.

## Appendix

**Table A1:** **This table illustrates the ranking of informative regions of interest (ROIs) based on the maximum magnitude of *t*-statistic across the three contrasts**.

Region of interest	LTLE vs. NES	RTLE vs. NES	LTLE vs. RTLE
Lilp temp C	**−3.974*****	0.158	**−3.410****
Lila temp C	**−3.493****	0.410	**−3.463*****
L sensorimotor C	0.230	**3.459*****	**−2.469***
Rila temp C	0.080	**−3.441****	**3.172****
Rpm temp C	2.583*	**−**1.147	**3.266****
Rs parietal C	**3.202****	0.631	**2.229***
R assoc. visual C	**2.641***	**−**1.128	**3.180****
Rsl temp C	1.450	**−2.343***	**2.995****
Lam temp C	**−**1.349	1.415	**−2.981****
Ram temp C	**2.176***	**−**1.234	**2.986****
Ri parietal C	**2.975****	1.266	**2.072***
R parietotemporal C	**2.246***	**−**0.325	**2.720****
Ls frontal C	**−**1.828	0.545	**−2.389***
Rilp temp C	0.447	**−2.321***	**2.361***
La cingulate C	1.274	**2.320***	**−**1.021
L thalamus	**−**1.663	**−**0.192	**−2.236***
Lpm temp C	**−**1.212	1.069	**−2.189***
Ri frontal C	**−**0.742	**−2.172***	1.409
Lsl temp C	**−**1.664	1.000	**−2.163***
L lentiform nucleus	**−**1.115	1.399	**−2.032***
Lm frontal C	**−**1.949	0.244	**−2.001***
Li frontal C	**−**1.930	0.059	**−**1.971
Ls frontal C	1.536	**−**0.351	1.943
Rm frontal C	1.482	**−**0.280	1.807
Rm frontal C	0.878	**−**0.883	1.726
Rp cingulate C	0.034	**−**1.215	1.712
R sensorimotor C	1.629	0.989	0.750
Li parietal C	1.576	1.361	0.193
R Broca’s region	1.108	**−**0.442	1.570
R caudate nucleus	1.462	0.925	0.559
L caudate nucleus	**−**0.937	0.599	**−**1.460
L Broca’s region	**−**1.028	0.668	**−**1.427
R primary visual C	**−**0.237	**−**1.362	1.393
Lm frontal C	1.096	1.370	**−**0.291
Ra cingulate cortex	1.286	0.407	0.889
R thalamus	0.266	**−**0.944	1.282
R lentiform nucleus	0.947	0.442	0.271
L primary visual C	**−**0.944	**−**0.149	**−**0.810
L assoc visual C	**−**0.142	**−**0.861	0.660
Vermis	**−**0.197	0.507	**−**0.701
Pons	0.667	0.473	0.153
L parietotemporal C	**−**0.569	**−**0.530	**−**0.028
R cerebellum	**−**0.087	0.406	**−**0.535
Lp cingulate C	**−**0.211	**−**0.498	0.393
L cerebellum	**−**0.461	**−**0.321	**−**0.054
Midbrain	**−**0.383	0.100	**−**0.435
Ls parietal cortex	0.093	0.195	**−**0.096

*Negative *t*-values in the first two columns indicate the hypometabolism in epilepsy. Negative *t*-values in the last column indicate hypometabolism in LTLE compared to RTLE. The leading L or R indicates left or right. The lowercase letters indicate inferior (i), lateral (l), median (m), anterior (a), and posterior (p). The lagging C signifies cortex. Bold indicates significant differences at greater than the 95% confidence level. The markings of *, **, and *** indicate significance at the 95, 99, and 99.9% confidence level, respectively, without multiple testing correction. No *t*-statistics remain significant at the 95% confidence level after Bonferroni correction*.

## References

[B1] Arnold S. Schlaug G. Niemann H. Ebner A. Luders H. Witte O. W. (1996). Topography of interictal glucose hypometabolism in unilateral mesiotemporal epilepsy. Neurology 46, 1422–1430 10.1212/WNL.46.5.1422 8628493

[B2] Barrick T. R. Mackay C. E. Prima S. Maes F. Vandermeulen D. Crow T. J. (2005). Automatic analysis of cerebral asymmetry: an exploratory study of the relationship between brain torque and planum temporale asymmetry. Neuroimage 24, 678–691 10.1016/j.neuroimage.2004.09.003 15652303

[B3] Barrington S. F. Koutroumanidis M. Agathonikou A. Marsden P. K. Binnie C. D. Polkey C. E. (1998). Clinical value of “ictal”. Epilepsia 39, 753–766 10.1111/j.1528-1157.1998.tb01162.x 9670905

[B4] Benbadis S. R. Tatum W. O. T. Murtagh F. R. Vale F. L. (2000). MRI evidence of mesial temporal sclerosis in patients with psychogenic nonepileptic seizures. Neurology 55, 1061–1062 10.1212/WNL.55.9.1421-a 11061274

[B5] Benedek K. Juhasz C. Muzik O. Chugani D. C. Chugani H. T. (2004). Metabolic changes of subcortical structures in intractable focal epilepsy. Epilepsia 45, 1100–1105 10.1111/j.0013-9580.2004.43303.x 15329075

[B6] Bernasconi A. Bernasconi N. Bernhardt B. C. Schrader D. (2011). Advances in MRI for ‘cryptogenic’ epilepsies. Nat. Rev. Neurol. 7, 99–108 10.1038/nrneurol.2010.199 21243016

[B7] Blum D. E. Ehsan T. Dungan D. Karis J. P. Fisher R. S. (1998). Bilateral temporal hypometabolism in epilepsy. Epilepsia 39, 651–659 10.1111/j.1528-1157.1998.tb01434.x 9637608

[B8] Bouckaert R. R. Frank E. Hall M. A. Holmes G. Pfahringer B. Ruetemann P. (2010). Weka-experiences with a java open-source project. J. Mach. Learn. Res. 11, 2533–2541

[B9] Bouilleret V. Dupont S. Spelle L. Baulac M. Samson Y. Semah F. (2002). Insular cortex involvement in mesiotemporal lobe epilepsy: a positron emission tomography study. Ann. Neurol. 51, 202–208 10.1002/ana.10087 11835376

[B10] Brodbeck V. Spinelli L. Lascano A. M. Pollo C. Schaller K. Vargas M. I. (2010). Electrical source imaging for presurgical focus localization in epilepsy patients with normal MRI. Epilepsia 51, 583–591 10.1111/j.1528-1167.2010.02521.x 20196796

[B11] Carne R. P. O’Brien T. J. Kilpatrick C. J. MacGregor L. R. Hicks R. J. Murphy M. A. (2004). MRI-negative PET-positive temporal lobe epilepsy: a distinct surgically remediable syndrome. Brain 127, 2276–2285 10.1093/brain/awh257 15282217

[B12] Chandra P. S. Salamon N. Huang J. Wu J. Y. Koh S. Vinters H. V. (2006). FDG-PET/MRI coregistration and diffusion-tensor imaging distinguish epileptogenic tubers and cortex in patients with tuberous sclerosis complex: a preliminary report. Epilepsia 47, 1543–1549 10.1111/j.1528-1167.2006.00627.x 16981871

[B13] Chauvin A. Worsley K. J. Schyns P. G. Arguin M. Gosselin F. (2005). Accurate statistical tests for smooth classification images. J. Vis. 5, 659–667 10.1167/5.9.1 16356076

[B14] Chinchure S. Kesavadas C. Thomas B. (2010). Structural and functional neuroimaging in intractable epilepsy. Neurol. India 58, 361–370 10.4103/0028-3886.65569 20644262

[B15] Cragar D. E. Berry D. T. Fakhoury T. A. Cibula J. E. Schmitt F. A. (2002). A review of diagnostic techniques in the differential diagnosis of epileptic and nonepileptic seizures. Neuropsychol. Rev. 12, 31–64 10.1023/A:1015491123070 12090718

[B16] Dabbs K. Becker T. Jones J. Rutecki P. Seidenberg M. Hermann B. (2012). Brain structure and aging in chronic temporal lobe epilepsy. Epilepsia 53, 1033–1043 10.1111/j.1528-1167.2012.03447.x 22471353 PMC3710695

[B17] Debets R. M. Sadzot B. Van Isselt J. W. Brekelmans G. J. Meiners L. C. Van Huffelen A. O. (1997). Is 11C-flumazenil PET superior to 18FDG PET and 123I-iomazenil SPECT in presurgical evaluation of temporal lobe epilepsy? J. Neurol. Neurosurg. Psychiatr. 62, 141–150 10.1136/jnnp.62.2.141 9048714 PMC486725

[B18] Delaney R. C. Rosen A. J. Mattson R. H. Novelly R. A. (1980). Memory function in focal epilepsy: a comparison of non-surgical, unilateral temporal lobe and frontal lobe samples. Cortex 16, 103–117 6769639 10.1016/s0010-9452(80)80026-8

[B19] Ding C. Peng H. C. (2005). Minimum redundancy feature selection from microarray gene expression data. J. Bioinform. Comput. Biol. 3, 185–205 10.1142/S0219720005001004 15852500

[B20] Dlugos D. J. Jaggi J. O’Connor W. M. Ding X. S. Reivich M. O’Connor M. J. (1999). Hippocampal cell density and subcortical metabolism in temporal lobe epilepsy. Epilepsia 40, 408–413 10.1111/j.1528-1157.1999.tb00734.x 10219265

[B21] Drzezga A. Arnold S. Minoshima S. Noachtar S. Szecsi J. Winkler P. (1999). 18F-FDG PET studies in patients with extratemporal and temporal epilepsy: evaluation of an observer-independent analysis. J. Nucl. Med. 40, 737–746 10319744

[B22] Duncan J. (2009). The current status of neuroimaging for epilepsy. Curr. Opin. Neurol. 22, 179–184 19300096 10.1097/WCO.0b013e328328f260

[B23] Engel J. Jr. McDermott M. P. Wiebe S. Langfitt J. T. Stern J. M. Dewar S. (2012). Early surgical therapy for drug-resistant temporal lobe epilepsy: a randomized trial. JAMA 307, 922–930 10.1001/jama.2012.220 22396514 PMC4821633

[B24] Farid N. Girard H. M. Kemmotsu N. Smith M. E. Magda S. W. Lim W. Y. (2012). Temporal lobe epilepsy: quantitative MR volumetry in detection of hippocampal atrophy. Radiology 264, 542–550 10.1148/radiol.12112638 22723496 PMC3401351

[B25] Focke N. K. Yogarajah M. Symms M. R. Gruber O. Paulus W. Duncan J. S. (2012). Automated MR image classification in temporal lobe epilepsy. Neuroimage 59, 356–362 10.1016/j.neuroimage.2011.07.068 21835245

[B26] Gaillard W. D. Bhatia S. Bookheimer S. Y. Fazilat S. Sato S. Theodore W. H. (1995). FDG-PET and volumetric MRI in the evaluation of patients with partial epilepsy. Neurology 45, 123–126 10.1212/WNL.45.10.1841 7824101

[B27] Gilbert D. L. Sethuraman G. Kotagal U. Buncher R. (2003). Meta-analysis of EEG test performance shows wide variation among studies. Neurology 60, 564–570 10.1212/01.WNL.0000044058.64647.7E 12601093

[B28] Haneef Z. Lenartowicz A. Yeh H. J. Engel J. Jr. Stern J. M. (2012). Effect of lateralized temporal lobe epilepsy on the default mode network. Epilepsy Behav. 25, 350–357 10.1016/j.yebeh.2012.07.019 23103309 PMC4209897

[B29] Henry T. R. Chupin M. Lehericy S. Strupp J. P. Sikora M. A. Sha Z. Y. (2011). Hippocampal sclerosis in temporal lobe epilepsy: findings at 7 T. Radiology 261, 199–209 10.1148/radiol.11101651 21746814 PMC3176424

[B30] Henry T. R. Mazziotta J. C. Engel J. Jr. (1993). Interictal metabolic anatomy of mesial temporal lobe epilepsy. Arch. Neurol. 50, 582–589 10.1001/archneur.1993.00540060022011 8503794

[B31] Henry T. R. Mazziotta J. C. Engel J. Jr. Christenson P. D. Zhang J. X. Phelps M. E. (1990). Quantifying interictal metabolic activity in human temporal lobe epilepsy. J. Cereb. Blood Flow Metab. 10, 748–757 10.1038/jcbfm.1990.128 2384546

[B32] Henry T. R. Roman D. D. (2011). Presurgical epilepsy localization with interictal cerebral dysfunction. Epilepsy Behav. 20, 194–208 10.1016/j.yebeh.2010.12.008 21257351

[B33] Henry T. R. Ross D. A. Schuh L. A. Drury I. (1999). Indications and outcome of ictal recording with intracerebral and subdural electrodes in refractory complex partial seizures. J. Clin. Neurophysiol. 16, 426–438 10.1097/00004691-199909000-00004 10576225

[B34] Iturria-Medina Y. Perez Fernandez A. Morris D. M. Canales-Rodriguez E. J. Haroon H. A. Garcia Penton L. (2011). Brain hemispheric structural efficiency and interconnectivity rightward asymmetry in human and nonhuman primates. Cereb. Cortex 21, 56–67 10.1093/cercor/bhq058 20382642

[B35] Juhasz C. Nagy F. Watson C. Da Silva E. A. Muzik O. Chugani D. C. (1999). Glucose and [11C]flumazenil positron emission tomography abnormalities of thalamic nuclei in temporal lobe epilepsy. Neurology 53, 2037–2045 10.1212/WNL.53.9.2037 10599778

[B36] Jung da E. Lee J. S. (2010). Multimodal neuroimaging in presurgical evaluation of childhood epilepsy. Korean J. Pediatr. 53, 779–785 10.3345/kjp.2010.53.8.779 21189974 PMC3004492

[B37] Jupp B. Williams J. Binns D. Hicks R. J. Cardamone L. Jones N. (2012). Hypometabolism precedes limbic atrophy and spontaneous recurrent seizures in a rat model of TLE. Epilepsia 53, 1233–1244 10.1111/j.1528-1167.2012.03525.x 22686573

[B38] Keihaninejad S. Heckemann R. A. Gousias I. S. Hajnal J. V. Duncan J. S. Aljabar P. (2012). Classification and lateralization of temporal lobe epilepsies with and without hippocampal atrophy based on whole-brain automatic MRI segmentation. PLoS ONE 7:e33096 10.1371/journal.pone.0033096 22523539 PMC3327701

[B39] Kerr W. T. Anderson A. Lau E. P. Cho A. Y. Xia H. Bramen J. (2012a). Automated diagnosis of epilepsy using EEG power spectrum. Epilepsia 53, e189–192 10.1111/j.1528-1167.2011.03326.x 22967005 PMC3447367

[B40] Kerr W. T. Anderson A. Xia H. Braun E. S. Lau E. P. Cho A. Y. (2012b). 2nd International Workshop Pattern Recognition in Neuroimaging. London: Conference Publishing Services 10.1109/PRNI.2012.27 PMC4169072 25241830

[B41] Kerr W. T. Lau E. P. Owens G. E. Trefler A. (2012c). The future of medical diagnostics: large digitized databases. Yale J. Biol. Med. 85, 363–377 23012584 PMC3447200

[B42] Kerr W. T. Lau E. P. (2012). Poisson noise obscures hypometabolic lesions in PET. Yale J. Biol. Med. 85, 541–549 23239953 PMC3516894

[B43] Kim H. Yi S. Son E. I. Kim J. (2003). Differential effects of left versus right mesial temporal lobe epilepsy on Wechsler intelligence factors. Neuropsychology 17, 556–565 10.1037/0894-4105.17.1.59 14599269

[B44] Kim Y. H. Kang H. C. Kim D. S. Kim S. H. Shim K. W. Kim H. D. (2011). Neuroimaging in identifying focal cortical dysplasia and prognostic factors in pediatric and adolescent epilepsy surgery. Epilepsia 52, 722–727 10.1111/j.1528-1167.2010.02950.x 21275980

[B45] Knowlton R. C. Elgavish R. A. Bartolucci A. Ojha B. Limdi N. Blount J. (2008). Functional imaging: II. Prediction of epilepsy surgery outcome. Ann. Neurol. 64, 35–41 10.1002/ana.21389 18570291

[B46] Knowlton R. C. Laxer K. D. Ende G. Hawkins R. A. Wong S. T. Matson G. B. (1997). Presurgical multimodality neuroimaging in electroencephalographic lateralized temporal lobe epilepsy. Ann. Neurol. 42, 829–837 10.1002/ana.410420603 9403474 PMC2709486

[B47] Kucyi A. Moayedi M. Weissman-Fogel I. Hodaie M. Davis K. D. (2012). Hemispheric asymmetry in white matter connectivity of the temporoparietal junction with the insula and prefrontal cortex. PLoS ONE 7:e35589 10.1371/journal.pone.0035589 22536413 PMC3334912

[B48] LaFrance W. C. Jr. Benbadis S. R. (2011). Differentiating frontal lobe epilepsy from psychogenic nonepileptic seizures. Neurol. Clin. 29, 149–162 10.1016/j.ncl.2010.10.005 21172576

[B49] LaFrance W. C. Jr. Devinsky O. (2004). The treatment of nonepileptic seizures: historical perspectives and future directions. Epilepsia 45(Suppl. 2), 15–21 10.1111/j.0013-9580.2004.452002.x 15186340

[B50] Lee E. M. Im K. C. Kim J. H. Lee J. K. Hong S. H. No Y. J. (2009). Relationship between hypometabolic patterns and ictal scalp EEG patterns in patients with unilateral hippocampal sclerosis: an FDG-PET study. Epilepsy Res. 84, 187–193 10.1016/j.eplepsyres.2009.02.005 19285834

[B51] Lee K. K. Salamon N. (2009). [18F] fluorodeoxyglucose–positron-emission tomography and MR imaging coregistration for presurgical evaluation of medically refractory epilepsy. AJNR Am. J. Neuroradiol. 30, 1811–1816 10.3174/ajnr.A1432 19628624 PMC7051291

[B52] Lerner J. T. Salamon N. Hauptman J. S. Velasco T. R. Hemb M. Wu J. Y. (2009). Assessment and surgical outcomes for mild type I and severe type II cortical dysplasia: a critical review and the UCLA experience. Epilepsia 50, 1310–1335 10.1111/j.1528-1167.2008.01998.x 19175385

[B53] Liao W. Zhang Z. Pan Z. Mantini D. Ding J. Duan X. (2011). Default mode network abnormalities in mesial temporal lobe epilepsy: a study combining fMRI and DTI. Hum. Brain Mapp. 32, 883–895 10.1002/hbm.21076 20533558 PMC6870458

[B54] Liew C. J. Lim Y. M. Bonwetsch R. Shamim S. Sato S. Reeves-Tyer P. (2009). 18F-FCWAY and 18F-FDG PET in MRI-negative temporal lobe epilepsy. Epilepsia 50, 234–239 10.1111/j.1528-1167.2008.01789.x 18801033 PMC2642908

[B55] Lin T. W. De Aburto M. A. Dahlbom M. Huang L. L. Marvi M. M. Tang M. (2007). Predicting seizure-free status for temporal lobe epilepsy patients undergoing surgery: prognostic value of quantifying maximal metabolic asymmetry extending over a specified proportion of the temporal lobe. J. Nucl. Med. 48, 776–782 10.2967/jnumed.107.042093 17475967

[B56] Liu R. S. Lemieux L. Bell G. S. Sisodiya S. M. Bartlett P. A. Shorvon S. D. (2002). The structural consequences of newly diagnosed seizures. Ann. Neurol. 52, 573–580 10.1002/ana.10408 12402254

[B57] Marchetti R. L. Kurcgant D. Gallucci Neto J. Von Bismark M. A. Fiore L. A. (2009). Evaluating patients with suspected nonepileptic psychogenic seizures. J. Neuropsychiatry Clin. Neurosci. 21, 292–298 10.1176/appi.neuropsych.21.3.292 19776309

[B58] Marchetti R. L. Kurcgant D. Neto J. G. Von Bismark M. A. Marchetti L. B. Fiore L. A. (2008). Psychiatric diagnoses of patients with psychogenic non-epileptic seizures. Seizure 17, 247–253 10.1016/j.seizure.2007.07.006 17702610

[B59] Matheja P. Kuwert T. Ludemann P. Weckesser M. Kellinghaus C. Schuierer G. (2001). Temporal hypometabolism at the onset of cryptogenic temporal lobe epilepsy. Eur. J. Nucl. Med. 28, 625–632 10.1007/s002590000352 11383869

[B60] McCormick C. Quraan M. Cohn M. Valiante T. A. McAndrews M. P. (2013). Default mode network connectivity indicates episodic memory capacity in mesial temporal lobe epilepsy. Epilepsia. [Epub ahead of print]. 10.1111/epi.12098 23360362

[B61] Morgan V. L. Rogers B. P. Sonmezturk H. H. Gore J. C. Abou-Khalil B. (2011). Cross hippocampal influence in mesial temporal lobe epilepsy measured with high temporal resolution functional magnetic resonance imaging. Epilepsia 52, 1741–1749 10.1111/j.1528-1167.2011.03196.x 21801166 PMC4428312

[B62] Morgan V. L. Sonmezturk H. H. Gore J. C. Abou-Khalil B. (2012). Lateralization of temporal lobe epilepsy using resting functional magnetic resonance imaging connectivity of hippocampal networks. Epilepsia 53, 1628–1635 10.1111/j.1528-1167.2012.03590.x 22779926 PMC3436984

[B63] Nelissen N. Van Paesschen W. Baete K. Van Laere K. Palmini A. Van Billoen H. (2006). Correlations of interictal FDG-PET metabolism and ictal SPECT perfusion changes in human temporal lobe epilepsy with hippocampal sclerosis. Neuroimage 32, 684–695 10.1016/j.neuroimage.2006.04.185 16762567

[B64] Peng H. Long F. Ding C. (2005). Feature selection based on mutual information: criteria of max-dependency, max-relevance, and min-redundancy. IEEE Trans. Pattern Anal. Mach. Intell. 27, 1226–1238 10.1109/TPAMI.2005.159 16119262

[B65] Pereira F. R. Alessio A. Sercheli M. S. Pedro T. Bilevicius E. Rondina J. M. (2010). Asymmetrical hippocampal connectivity in mesial temporal lobe epilepsy: evidence from resting state fMRI. BMC Neurosci. 11:66 10.1186/1471-2202-11-66 20525202 PMC2890013

[B66] Person C. Koessler L. Louis-Dorr V. Wolf D. Maillard L. Marie P. Y. (2010). Analysis of the relationship between interictal electrical source imaging and PET hypometabolism. Conf. Proc. IEEE Eng. Med. Biol. Soc. 2010, 3723–3726 21096861 10.1109/IEMBS.2010.5627512

[B67] Pittau F. Grova C. Moeller F. Dubeau F. Gotman J. (2012). Patterns of altered functional connectivity in mesial temporal lobe epilepsy. Epilepsia 53, 1013–1023 10.1111/j.1528-1167.2012.03464.x 22578020 PMC3767602

[B68] Privitera M. (2011). Current challenges in the management of epilepsy. Am. J. Manag. Care 17(Suppl. 7), S195–203 21761951

[B69] Rastogi S. Lee C. Salamon N. (2008). Neuroimaging in pediatric epilepsy: a multimodality approach. Radiographics 28, 1079–1095 10.1148/rg.284075114 18635630

[B70] Reuber M. Fernandez G. Helmstaedter C. Qurishi A. Elger C. E. (2002). Evidence of brain abnormality in patients with psychogenic nonepileptic seizures. Epilepsy Behav. 3, 249–254 10.1016/S1525-5050(02)00004-5 12662605

[B71] Reutens D. C. Stevens J. M. Kingsley D. Kendall B. Moseley I. Cook M. J. (1996). Reliability of visual inspection for detection of volumetric hippocampal asymmetry. Neuroradiology 38, 221–225 10.1007/BF00596533 8741191

[B72] Rusu V. Chassoux F. Landre E. Bouilleret V. Nataf F. Devaux B. C. (2005). Dystonic posturing in seizures of mesial temporal origin: electroclinical and metabolic patterns. Neurology 65, 1612–1619 10.1212/01.wnl.0000184510.44808.50 16301490

[B73] Ryvlin P. Cinotti L. Froment J. C. Le Bars D. Landais P. Chaze M. (1991). Metabolic patterns associated with non-specific magnetic resonance imaging abnormalities in temporal lobe epilepsy. Brain 114(Pt 6), 2363–2383 10.1093/brain/114.6.2363 1782521

[B74] Sadzot B. Debets R. M. Maquet P. Van Veelen C. W. Salmon E. Van Emde Boas W. (1992). Regional brain glucose metabolism in patients with complex partial seizures investigated by intracranial EEG. Epilepsy Res. 12, 121–129 10.1016/0920-1211(92)90032-O 1396538

[B75] Sahaya K. Dholakia S. A. Sahota P. K. (2011). Psychogenic non-epileptic seizures: a challenging entity. J. Clin. Neurosci. 18, 1602–1607 10.1016/j.jocn.2011.05.016 22051027

[B76] Salamon N. Kung J. Shaw S. J. Koo J. Koh S. Wu J. Y. (2008). FDG-PET/MRI coregistration improves detection of cortical dysplasia in patients with epilepsy. Neurology 71, 1594–1601 10.1212/01.wnl.0000334752.41807.2f 19001249 PMC2676967

[B77] Schmidt M. H. Pohlmann-Eden B. (2011). Neuroimaging in epilepsy: the state of the art. Epilepsia 52(Suppl. 4), 49–51 10.1111/j.1528-1167.2011.03154.x 21732944

[B78] Shehab S. Simkins M. Dean P. Redgrave P. (1995). The dorsal midbrain anticonvulsant zone – I. Effects of locally administered excitatory amino acids or bicuculline on maximal electroshock seizures. Neuroscience 65, 671–679 10.1016/0306-4522(94)00517-9 7609869

[B79] So E. L. O’Brien T. J. Brinkmann B. H. Mullan B. P. (2000). The EEG evaluation of single photon emission computed tomography abnormalities in epilepsy. J. Clin. Neurophysiol. 17, 10–28 10.1097/00004691-200001000-00003 10709808

[B80] Sperling M. R. Gur R. C. Alavi A. Gur R. E. Resnick S. O’Connor M. J. (1990). Subcortical metabolic alterations in partial epilepsy. Epilepsia 31, 145–155 10.1111/j.1528-1167.1990.tb06299.x 2108014

[B81] Struck A. F. Hall L. T. Floberg J. M. Perlman S. B. Dulli D. A. (2011). Surgical decision making in temporal lobe epilepsy: a comparison of [(18)F]FDG-PET, MRI, and EEG. Epilepsy Behav. 22, 293–297 10.1016/j.yebeh.2011.06.022 21798813 PMC3260654

[B82] Swartz B. E. Tomiyasu U. Delgado-Escueta A. V. Mandelkern M. Khonsari A. (1992). Neuroimaging in temporal lobe epilepsy: test sensitivity and relationships to pathology and postoperative outcome. Epilepsia 33, 624–634 10.1111/j.1528-1157.1992.tb02338.x 1628575

[B83] Takaya S. Hanakawa T. Hashikawa K. Ikeda A. Sawamoto N. Nagamine T. (2006). Prefrontal hypofunction in patients with intractable mesial temporal lobe epilepsy. Neurology 67, 1674–1676 10.1212/01.wnl.0000242628.26978.e2 17101904

[B84] Theodore W. H. Katz D. Kufta C. Sato S. Patronas N. Smothers P. (1990). Pathology of temporal lobe foci: correlation with CT, MRI, and PET. Neurology 40, 797–803 10.1212/WNL.40.5.797 2330107

[B85] Toga A. W. Thompson P. M. (2003). Mapping brain asymmetry. Nat. Rev. Neurosci. 4, 37–48 10.1038/nrn1009 12511860

[B86] Uijl S. G. Leijten F. S. Arends J. B. Parra J. Van Huffelen A. C. Moons K. G. (2007). The added value of [18F]-fluoro-D-deoxyglucose positron emission tomography in screening for temporal lobe epilepsy surgery. Epilepsia 48, 2121–2129 10.1111/j.1528-1167.2007.01197.x 17651417

[B87] van Ginneken B. Shaefer-Prokop C. M. Prokop M. (2011). Computer-aided diagnosis: how to move from the laboratory to the clinic. Radiology 261, 719–732 10.1148/radiol.11091710 22095995

[B88] Van Paesschen W. Duncan J. S. Stevens J. M. Connelly A. (1998). Longitudinal quantitative hippocampal magnetic resonance imaging study of adults with newly diagnosed partial seizures: one-year follow-up results. Epilepsia 39, 633–639 10.1111/j.1528-1157.1998.tb01432.x 9637606

[B89] Wang Y. Van Klaveren R. J. De Bock G. H. Zhao Y. Vernhout R. Leusveld A. (2012). No benefit for consensus double reading at baseline screening for lunch cancer with the use of semiautomated volumetry software. Radiology 262, 320–326 10.1148/radiol.11110470 22106357

[B90] Willmann O. Wennberg R. May T. Woermann F. G. Pohlmann-Eden B. (2007). The contribution of 18F-FDG PET in preoperative epilepsy surgery evaluation for patients with temporal lobe epilepsy A meta-analysis. Seizure 16, 509–520 10.1016/j.seizure.2007.04.001 17532231

[B91] Worsley K. J. Evans A. C. Marrett S. Neelin P. (1992). A three-dimensional statistical analysis for CBF activation studies in human brain. J. Cereb. Blood Flow Metab. 12, 900–918 10.1038/jcbfm.1992.127 1400644

[B92] Worsley K. J. Taylor J. E. Tomaiuolo F. Lerch J. (2004). Unified univariate and multivariate random field theory. Neuroimage 23, S189–S195 10.1016/j.neuroimage.2004.07.026 15501088

[B93] Yun C. H. Lee S. K. Lee S. Y. Kim K. K. Jeong S. W. Chung C. K. (2006). Prognostic factors in neocortical epilepsy surgery: multivariate analysis. Epilepsia 47, 574–579 10.1111/j.1528-1167.2006.00470.x 16529624

[B94] Zhang Z. Lu G. Zhong Y. Tan Q. Liao W. Wang Z. (2010). Altered spontaneous neuronal activity of the default-mode network in mesial temporal lobe epilepsy. Brain Res. 1323, 152–160 10.1016/j.brainres.2010.01.042 20132802

[B95] Zhou J. Gennatas E. D. Kramer J. H. Miller B. L. Seeley W. W. (2012). Predicting regional neurodegeneration from the healthy brain functional connectome. Neuron 73, 1216–1227 10.1016/j.neuron.2012.03.004 22445348 PMC3361461

